# Chondroitin Sulfate Synthase-2 Is Necessary for Chain Extension of Chondroitin Sulfate but Not Critical for Skeletal Development

**DOI:** 10.1371/journal.pone.0043806

**Published:** 2012-08-28

**Authors:** Hiroyasu Ogawa, Sonoko Hatano, Nobuo Sugiura, Naoko Nagai, Takashi Sato, Katsuji Shimizu, Koji Kimata, Hisashi Narimatsu, Hideto Watanabe

**Affiliations:** 1 Institute for Molecular Science of Medicine, Aichi Medical University, Nagakute, Japan; 2 Research Center for Medical Glycoscience, Advanced Industrial Science and Technology, Nagakute, Japan; 3 Department of Orthopaedic Surgery, Gifu University, Graduate School of Medicine, Gifu, Japan; 4 Research Complex for Medicine Frontiers, Aichi Medical University, Nagakute, Japan; University of Patras, Greece

## Abstract

Chondroitin sulfate (CS) is a linear polysaccharide consisting of repeating disaccharide units of *N-*acetyl-D-galactosamine and D-glucuronic acid residues, modified with sulfated residues at various positions. Based on its structural diversity in chain length and sulfation patterns, CS provides specific biological functions in cell adhesion, morphogenesis, neural network formation, and cell division. To date, six glycosyltransferases are known to be involved in the biosynthesis of chondroitin saccharide chains, and a hetero-oligomer complex of chondroitin sulfate synthase-1 (CSS1)/chondroitin synthase-1 and chondroitin sulfate synthase-2 (CSS2)/chondroitin polymerizing factor is known to have the strongest polymerizing activity. Here, we generated and analyzed CSS2^−/−^ mice. Although they were viable and fertile, exhibiting no overt morphological abnormalities or osteoarthritis, their cartilage contained CS chains with a shorter length and at a similar number to wild type. Further analysis using CSS2^−/−^ chondrocyte culture systems, together with siRNA of CSS1, revealed the presence of two CS chain species in length, suggesting two steps of CS chain polymerization; i.e., elongation from the linkage region up to Mr ∼10,000, and further extension. There, CSS2 mainly participated in the extension, whereas CSS1 participated in both the extension and the initiation. Our study demonstrates the distinct function of CSS1 and CSS2, providing a clue in the elucidation of the mechanism of CS biosynthesis.

## Introduction

Chondroitin sulfate (CS) is a linear polysaccharide consisting of repeating disaccharide units of *N-*acetyl-D-galactosamine (GalNAc) and D-glucuronic acid (GlcUA) residues, modified with sulfated residues at various positions [1]–[4]. CS chains exhibit structural diversity in chain length and sulfation patterns, providing specific biological functions in cell adhesion, morphogenesis, neural network formation, and cell division [5]–[10].

CS biosynthesis is initiated by transfer of GalNAc to the linkage region of a glucuronic acid- galactose-galactose-xylose (GlcUA-Gal-Gal-Xyl) tetrasaccharide primer that is attached to a serine residue of a core protein. Following this step termed chain initiation, chain polymerization occurs by the alternate addition of GalNAc and GlcUA residues. The enzymatic activities that catalyze these initiation and polymerization processes are designated glycosyltransferase-I and -II activities, respectively [4]. To date, six glycosyltransferases involved in mammalian CS biosynthesis have been identified: chondroitin sulfate synthase-1 (CSS1)/chondroitin synthase-1 (ChSy-1) [11]–[12], chondroitin sulfate synthase-2 (CSS2)/chondroitin polymerizing factor (ChPF) [12], [13], chondroitin sulfate synthase-3 (CSS3)/chondroitin synthase-2 (ChSy-2) [14], [15], chondroitin sulfate glucuronyltransferase (CSGlcAT)/chondroitin synthase-3 (ChSy-3) [16], [17], and chondroitin sulfate *N*-acetylgalactosaminyltransferase (CSGalNAcT) -1 [18] and -2 [19]. CSS1, CSS2, and CSS3 contain two glycosyltransferase domains: *β*-3 domain at the N-terminal region and *β*-4 domain at the C-terminal region. In addition, they exhibit dual enzymatic activities of *N*-acetylgalactosaminyltransferase-II (GalNAcT-II) and glucuronyltransferase-II (GlcAT-II); another research group failed to find glycosyltransferase activity in CSS2, however, thereby naming chondroitin polymerizing factor (ChPF). CSGlcAT, similarly containing two glycosyltransferase domains, shows only GlcAT-I activity [16], though another report revealed GalNAcT activity in CSGlcAT [17], thus renaming ChSy-3. CSGalNAcT-1 and -2 contain a *β*-4 glycosyltransferase domain, and exhibit both GalNAcT-I and -II activities responsible for chain initiation and polymerization, respectively [18], [19].

Biochemical characterization and expression profiles of these enzymes suggest their functional specificity and redundancy. CSS1 exhibits the highest glycosyltransferase activity, followed by CSS2, and then CSGlcAT and CSS3 [11], [13]. Similarly, CSS1 has the highest expression level in most tissues, followed by CSS2 and CSGlcAT, whereas CSS3 shows minimal expression levels [14]. Though CSS1, CSS2, and CSS3 exhibit both GalNAcT-II and GlcAT-II activities, co-expression of two of them is required to achieve the polymerization and any one of them or a mixture of two does not polymerize CS chains [17]. These *in vitro* results suggest that the CS polymerizing machinery is made up of at least the hetero-oligomer of two glycosyltransferases. Out of several combinations, that of CSS1 and CSS2 has been shown to exhibit the highest polymerization activity [15], [17]. These observations strongly suggest that the hetero-oligomer of CSS1 and CSS2 is the major player in CS biosynthesis. However, as the sample by co-expression of any two of the four enzymes achieves chain polymerization, and there are tissues where the expression levels of CSS1 and CSS2 are considerably low [15], the *in vivo* mechanism of CS biosynthesis remains elusive.

To address the *in vivo* mechanism of CS synthesis, we generated CSS2^−/−^ mice and analyzed them. Though CSS2/ChPF was initially reported to be critical for CS biosynthesis [13], CSS2^−/−^ mice were viable and fertile, exhibiting no overt abnormalities. Biochemically, their cartilage showed a reduction in CS chain length from 19,000 to 10,000 in molecular weight, with no difference in the number and sulfation profiles of CS, as compared with wild-type (WT) littermates. Further analysis using chondrocyte culture, where CSS1 expression was inhibited with siRNA, revealed that both CSS2 and CSS1 are necessary for CS chain polymerization, especially for extension from 10,000 to 19,000 in molecular weight, and that whereas CSS1 regulates both the chain initiation and the extension, CSS2 does for the extension. These results provide insight into the functional specificity and redundancy of glycosyltransferases involved in CS biosynthesis.

## Materials and Methods

### Materials

[^3^H]-sodium borohydride (2.96–3.7 TBq/mmol) was purchased from PerkinElmer Life Sciences. Chondroitinase ABC was from Seikagaku Biobusiness (Tokyo). Superose 6 HR 10/30 and Superose 12 HR 10/30 columns were purchased from GE Healthcare.

### Generation of CSS2^−/−^ Mice

This work was approved by the Animal Care Committee at Aichi Medical University (#2009-42). We generated CSS2^−/−^ mice, using both Cre/loxP and Flp/FRT systems as follows. A targeting vector harboring CSS2^flox^ allele was constructed by flanking exon 1 containing the putative start codon of the mouse CSS2 gene with loxP sites in combination with a neomycin selection (Neo) cassette flanked by the FRT sequence. Then, mouse ES cells were electroporated with pre-linearized targeting construct and cultured with G418 for positive selection. Clones were screened by genomic PCR, and their homologous recombination was confirmed by genomic Southern blotting. By blastocyst injection, chimeric mice were obtained. Germline transmission of CSS2^flox^ allele was attained by crossing these chimeric mice with C57BL6. Then, by crossing with CAG-flippase transgenic (Tg) mice [20], CSS2^+/flox^ mice, whose genome lacked the Neo cassette, were obtained. Then by crossing CSS2^+/flox^ mice with CAG-Cre Tg mice [21] with the background of C57BL6, CAG-Cre/CSS2^+/−^ mice were obtained. CAG-Cre/CSS2^+/−^ male and female mice were crossed to obtain CAG-Cre/CSS2^−/−^ mice whose CSS2 gene was removed by Cre-mediated excision (CSS2^−/−^). Genotyping of mice was performed by PCR using *KOD*-Plus DNA polymerase (TOYOBO), genomic DNA from a tail biopsy as template, and primers: 5′-GTGTAGAAGCATACGGCATAGTGG-3′ and 5′-AGTGCCTGATACCTGCGCTCCAGAGG-3′, with a program of 35 cycles at 98°C for 10 s, 67°C for 30 s, and 72°C for 180 s, generating a PCR product of 2.1 kb in the WT allele and 420 bp in the mutant allele, respectively. The sequence analysis of the PCR product was performed using an ABI PRISM® 3130 Genetic Analyzer (Applied Biosystems) to confirm the lack of exon 1 of CSS2 in genomic DNA.

### Culture of Chondrocytes

Rib chondrocytes were prepared from 2-day-old WT and CSS2^−/−^ mice, as described previously with slight modification [22]. In brief, the rib cartilage was incubated with 0.25% trypsin for 15 min at 37°C, and then with 0.2% type II collagenase (Wako, Osaka) in Dulbecco’s modified Eagle’s medium (DMEM, Sigma-Aldrich) for 90 min, and rinsed with PBS to remove soft tissues. The sample was digested with 0.2% type II collagenase in DMEM for 4 h, and chondrocytes released from the tissue were obtained. The cells were maintained in DMEM containing 10% (v/v) fetal bovine serum (FBS), 50 µg/ml streptomycin, and 50 units/ml penicillin, and were plated on culture dishes at a density of 5.0×10^4^ cells/cm^2^. All experiments were performed using primary chondrocytes.

### Histological Analysis of Skeleton and Organs

For the skeletal analysis, the whole skeletons of newborn and 1-month-old mice were fixed in 96% ethanol, stained in a solution containing Alcian blue 8GX (Sigma-Aldrich) for 3 days, dehydrated in 100% ethanol for 5 days, immersed in 1% KOH solution for 3 days, stained in 1% KOH solution containing Alizarin red S (Sigma-Aldrich) for 2 days, and stored in 100% glycerol. The longitudinal lengths of humerus, ulna, femur, and tibia of WT and CSS2^−/−^1-month-old mice were measured with a light microscope and NIH image using 10 stained samples each. Statistical analyses were calculated by Student’s *t* test. For histological analysis of organs, skin, brain, heart, lung, liver, spleen, kidney, small intestine of 1 month-old mice were fixed in 4% paraformaldehyde at 4°C overnight, and embedded in paraffin. For the examination of cartilage growth plate, 8 proximal humeri were decalcified in K-CX (Farma) for 24 h at room temperature before paraffin embedding. The paraffin blocks were sectioned at 8-µm thickness and stained using hematoxylin and eosin. For the examination of osteoarthritis, knee joints (n = 8, each) were harvested from WT and CSS2^−/−^ mice at the ages of 1 and 6 months, respectively. All the joints were fixed in 4% paraformaldehyde and processed as described above. For each knee joint, 8 µm-thick serial sections were cut from a lateral-to-medial direction of the joints. Approximately 100 sections covered an entire knee joint from its anterior to its posterior direction with meniscus. Every tenth section was collected for safranin O and fast green staining. The section slides were histologically examined under a light microscope, BZ-8000 (Keyence, Osaka, Japan).

### Immunoprecipitation of CSS2

Mouse embryonic fibroblasts (MEFs), were obtained from E14.5 embryos as described previously [23], and cultured in DMEM containing 10% fetal bovine serum (FBS), penicillin, and streptomycin. The cells at passage 4 were grown up to the confluence on twenty 15-cm culture dishes, and collected and suspended in 40 ml cell lysis buffer (10 mM Tris-HCl pH 7.4, 1.5 mM EDTA, 140 mM NaCl, 1% Triton X-100, 25 mM NaF, with freshly added proteinase inhibitor cocktail). The lysate was incubated for 1 h at 4°C in a rotation shaker, and clarified by centrifugation (14,000 rpm for 30 min at 4°C). The supernatant of the lysate was pre-cleared with 30 µl Protein G Sepharose™ 4 Fast Flow (GE Healthcare) for 2 h at 4°C in a rotation shaker, and then incubated overnight at 4°C in a rotation shaker with 20 µg of an anti-CSS2 antibody pre-bound to 30 µl Protein G Sepharose™ 4 Fast Flow [24]. The beads were recovered by centrifugation and washed five times with PBS, and then subjected to western blot analysis.

### Western Blot Analysis

The cell lysates obtained from MEFs were separated by 10% SDS-PAGE, and proteins were electrotransferred to a polyvinylidene difluoride membrane. After blocking for 1 h in 20 mM Tris-HCl pH 7.4, 150 mM NaCl (TBS) containing 5% skim milk and 0.1% Tween 20 at room temperature, the membrane was incubated with an anti-CSS2 antibody [24] at 4°C overnight. After washing with TBS for 10 min three times, the membrane was incubated with a goat anti-rabbit antibody conjugated with horseradish peroxidase (Cappel) for 1 h at room temperature. After washing with TBS as above, proteins bound to the antibodies were visualized with SuperSignal® West Femto Maximum Sensitivity Substrate (Thermo Scientific) according to the manufacturer’s instruction.

### Quantitative Analysis of CSS1, CSS2, CSGlcAT/ChSy3, Csgalnact1, and Csgalnact2 by Realtime RT-PCR

Total RNAs were isolated from fresh humerus of newborn mice using an RNeasy Mini kit (Qiagen), and cDNA templates were synthesized from the total RNA with a QuantiTect Reverse Transcription kit (Qiagen). Realtime PCR was performed, using the cDNAs, Power SYBR Green PCR Master Mix (Applied Biosystems), and primers specific for CSS1, CSS2, CSGlcAT/ChSy3, Csgalnact1, and Csgalnact2 (Table 1) with StepOnePlus Real-Time PCR System (Applied Biosystems) according to the manufacturer’s protocols. Each reaction was performed in a triplicate in three independent experiments. The relative amounts of the transcripts were normalized with the amount of GAPDH transcript in the same cDNA samples using TaqMan Rodent GAPDH Control Reagents VIC (Applied Biosystems).

**Table 1:** Primers for realtime PCR.

Gene	Primer
CSS1	5′-CTGTAACCCTGGTCGATGCTGA-3′
	5′-GTTTGTGCAAAGGTGACAGGTGA-3′
CSS2	5′-TTCGTCCCTCTCCGCTAGCTGACG-3′
	5′-AAGGCGGCCGCTGTCCGACGTGTC-3′
Chsy3	5′-ACTGGGTGCAGGTGGATTTGATA-3′
	5′-GGCCGTAAGCCAGATAGGATGA-3′
Csgalnact1	5′-TCTCTGGTTCCTGCTCACTGAAATA-3′
	5′-TCTGCTTGGTCTGAGACTTTGGTAG-3′
Csgalnact2	5′-TCTGCCTGGTACCTGTGTTCTGA-3′
	5′-TATCTGCCTGAACATTTCGACCAA-3′

### Extraction of Glycosaminoglycans

Minced proximal humerus of newborn mice and cultured chondrocytes were used as the source of glycosaminoglycans (GAGs). GAGs were released from the core protein with 0.2 M NaOH for 16 h at room temperature. The samples were neutralized by the addition of 4 M acetate, treated with DNase and RNase for 2 h at 37°C, and digested with 1 mg/ml proteinase K in 50 mM Tris-HCl, pH 8.0 for 2 h at 56°C. The samples were centrifuged and the supernatants were applied to a DEAE-Sephacel (Amersham Biosciences) column equilibrated with 50 mM Tris-HCl, pH 7.5. After washing with 10 column volumes of 50 mM Tris-HCl, pH 7.5, 0.2 M NaCl, GAG-rich fractions were eluted with 50 mM Tris-HCl, pH 7.5, 2 M NaCl. The eluates were precipitated by the addition of 3 volumes of 95% ethanol containing 1.3% potassium acetate, and the precipitated GAGs were dissolved in distilled water.

### Structural and Quantitative Analysis of CS

To examine the disaccharide composition of CS, an aliquot of the extracted GAGs as above was removed of low molecular weight proteins and small oligosaccharides by filtration using Ultrafree-MC (5,000 molecular weight limit, Millipore). The GAGs not filtrated were treated with 30 milliunits of chondroitinase ABC in 25 µl of 50 mM Tris-HCl, pH 7.5, 0.04% bovine serum albumin for 2 h at 37°C, and filtered using Ultrafree-MC (5,000 molecular weight limit) again. Unsaturated disaccharides in the filtrates were analyzed according to a previously described method [25] with a slight modification of elution conditions. To examine the CS chain length, an aliquot of the isolated GAGs was treated with the heparitinase mixture and labeled with [^3^H]-sodium borohydride. Briefly, 10 µl of sample source was reacted with 8.4 pmol of [^3^H]-sodium borohydride (12 nCi/pmol) for 3 h at room temperature, treated with 2 µl of 2M CH_3_COOH, and neutralized with 2 µl of 2M NaOH. After removal of free [^3^H]-sodium borohydride by precipitation using 3 volumes of 95% ethanol containing 1.3% potassium acetate, the labeled sample was applied to a Superose 6 or 12 column equilibrated in 0.2 M NaCl, followed by scintillation counting of each fraction. To estimate the relative number of CS chains, the total radioactivity of elution profile was calculated. Radioactivity was expressed per mg on cell- or tissue-protein, which was quantified after the NaOH treatment using Micro BCA™ Protein Assay Kit (Pierce). Molecular weight standards described previously [24] were used.

### RNA Interference of the CSS1 Gene

Stealth RNAi oligonucleotides (Invitrogen) were used for siRNA experiments. The following sequences were used for CSS1: antisense, 5′-UAUAGAUGUAUCUGAACCCUCGCUA-3′; and sense, 5′-UAGCGAGGGUUCAGAUACAUCUAUA-3′. For a negative control, a low GC duplex of Stealth RNAi negative control duplexes (Invitrogen) was used. The Stealth RNAi oligonucleotides were transfected into cells using Lipofectamine RNAiMAX (Invitrogen) according to the manufacturer’s protocols. Non-transfected cells were used as mock. After 48 h, the cells were subjected to realtime RT-PCR and CS analysis.

## Results

### Generation of CSS2^−/−^ Mice

**Figure 1:**
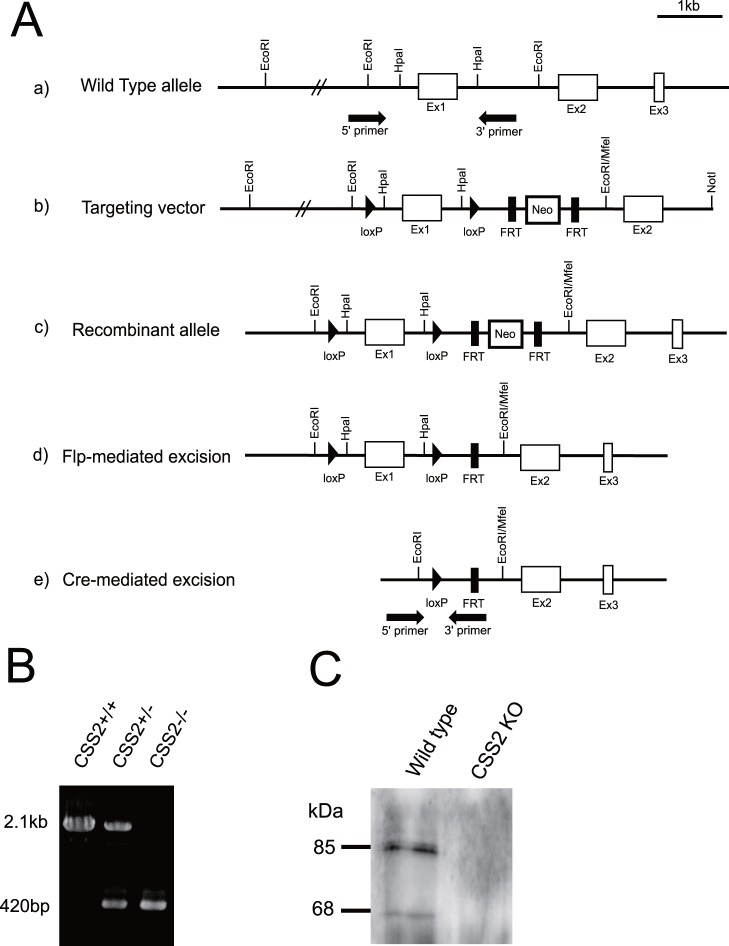
Generation of CSS2^−/−^ mice. A, Genomic structure of CSS2 gene, the targeting vector, and the genome with homologous recombination and that whose fragments were excised by flippase and Cre systems. *A*, The genomic structure of CSS2 gene is depicted on the top line (a). CSS2 targeting vector (b) was constructed by flanking exon 1 with *loxP* sites, and flanking a Neo^R^ cassette with FRT sites. ES cell clones with homologous recombination of the vector segment (c) were obtained by positive selection, and used for generation of chimera mice, followed by germ line transmission. The FRT-flanked Neo cassette was subsequently deleted from the recombinant allele by crossing with CAG-Flp Tg mice (d). Then, a genomic fragment containing exon 1, flanked by loxP sites, was excised from the recombinant allele by crossing with CAG-Cre Tg mice (e). *B*, Genomic PCR. Genotyping was performed by PCR using tail DNA and primers shown in black arrows in Fig. 1A. The PCR product of wild-type allele and CSS2 mutant allele was 2.1 kb and 420 bp respectively. *C*, Immunoprecipitation of the CSS2, followed by western blot. The cell lysates obtained from WT MEFs were subjected to western blot analysis as described in “Experimental Procedures”. Western blot analysis shows bands of CSS2 (85 kDa) and the CSS2 variant (68 kDa).

We designed a conditional targeting vector for CSS2, in which exon 1 was flanked with the loxP sequence, while a Neo cassette flanked with the FRT sequence was inserted in intron 1 (Fig. 1A). Chimeric mice were obtained by blastocyst injection of ES cell clones with homologous recombination. They were crossed with WT mice, and offspring with germline transmission were obtained. Then, we crossed these mice with CAG-flippase Tg mice and obtained CAG-flippase Tg/CSS2^+/flox^ mice whose genomic DNA lacks the NeoR cassette. These mice were crossed with C57BL6 mice to segregate the CAG-flippase transgene. Then, by crossing CSS2^+/flox^ male and female mice, we obtained CSS2^flox/flox^ mice. CSS2^+/flox^ and CSS2^flox/flox^ mice were healthy and fertile. Next, we crossed CSS2^+/flox^ mice with CAG-Cre Tg mice and obtained CAG-Cre/CSS2^+/−^ mice, which were crossed with C57BL6 mice to segregate the CAG-Cre transgene. Then, by crossing these mice, we obtained CSS2^−/−^ mice, which lack CSS2 expression in the entire body. Their genotype was determined by genomic PCR, and the sequence analysis of the PCR products showed that Cre recombinase efficiently ablated exon 1 of CSS2 in the genome. In genomic PCR, the bands at 2.1 kb and 420 bp represent the WT and CSS2 mutant alleles, respectively (Fig. 1B). Immunoprecipitation and following western blot analysis detected CSS2, as previously reported [24]. The sample from the WT mice showed two bands at 85 kDa and 68 kDa corresponding to original CSS2, i.e., CSS2A, and the CSS2 variant, CSS2B [24], respectively. In contrast, that from CSS2^−/−^ mice showed no bands (Fig. 1C), indicating that CSS2^−/−^ mice express neither CSS2A nor CSS2B.

### CSS2^−/−^ Mice Exhibit No Overt Morphological Phenotype

**Figure 2:**
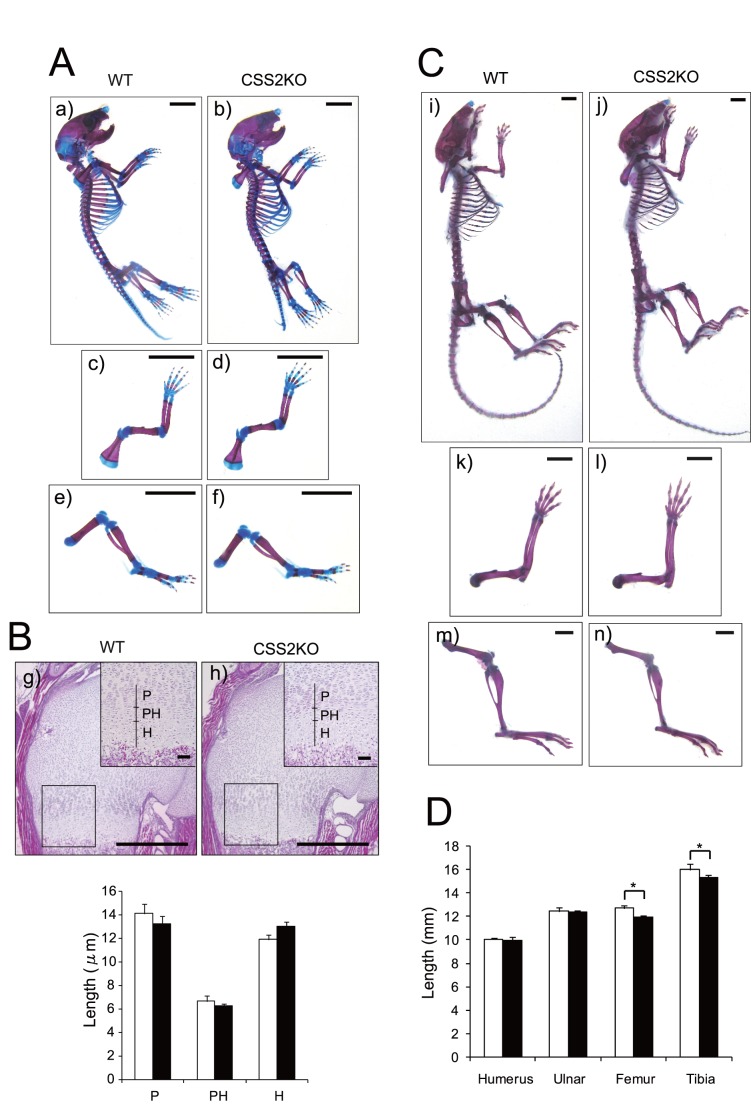
Skeletal analysis of CSS2^−/−^ (CSS2KO) mice and their wild-type (WT) littermates at newborn and age of 1 month. *A*, skeletal structure of CSS2KO and WT newborn mice stained with alcian blue and alizarin red. Whole body (a, b), upper extremity (c, d), and lower extremity (e, f) of WT littermates and CSS2KO mice, respectively, were shown. Scale bar: 500 µm. *B*, Proximal humerus of WT and CSS2KO newborn mice stained with hematoxylin-eosin is shown. The area size, the number and orientation of chondrocytes are similar between WT littermates (g) and CSS2KO (h) mice. The lengths of proliferative (P), prehypertrophic (PH), and hypertrophic (H) zones of WT (□) littermates and CSS2KO (▪) mice are shown in the histogram. Bar graph shown in the graphs indicate a mean ± S.D. of 10 samples’ measurements (n = 10). Large scale bar: 50 µm, small scale bar: 5 µm. *C*, skeletal structure of CSS2KO and WT 1-month-old mice stained with alcian blue and alizarin red. Whole body (i, j), upper extremity (k, l), and lower extremity (m, n) of WT littermates and CSS2KO mice, respectively, were shown. Scale bar: 5 mm. *D*, The histogram shows comparison of the length of humerus, ulna, femur, and tibia of WT (□) littermates and CSS2KO (▪) mice. Bar graph shown in the graphs indicate a mean ± S.D. of 10 samples’ measurements (n = 10). *; p<0.005.

General examinations, including body size and weight, showed no significant phenotypic differences between WT and CSS2^−/−^ mice. Then, the skin, brain, heart, lung, liver, spleen, kidney, small intestine of each of three WT and CSS2^−/−^ mice were histologically screened for phenotype using HE staining. CSS2^−/−^ mice were indistinguishable from their littermate controls based on gross and histological appearances of these tissues (Fig. 1). Next, detailed analysis of the skeletal structure and growth plate of the proximal humerus of CSS2^−/−^ mice was performed, as CS is abundant in cartilage, playing important roles in cartilage growth plate morphogenesis and mammalian skeletal development [26]. To examine skeletal development, we measured the bone length of WT and CSS2^−/−^ newborn and one-month-old mice (five mice each). When skeletal preparations of humerus double-stained with Alizarin red and Alcian blue were observed, there were no clear gross differences between WT and CSS2^−/−^ mice at newborn and one month (Fig. 2A and 2C). When measured, while the length of humerus, ulna, femur, and tibia of newborn mice was similar between WT and CSS2^−/−^ (data not shown), that of femur and tibia of one-month-old CSS2^−/−^ mice was slightly reduced, compared with that of WT mice (Fig. 2D). The growth plate of the proximal humerus of CSS2^−/−^ newborn mice contained well-organized chondrocyte columns, and the populations of the proliferative, prehypertrophic, and hypertrophic chondrocytes were similar to those of WT mice (Fig. 2B).

**Figure 3:**
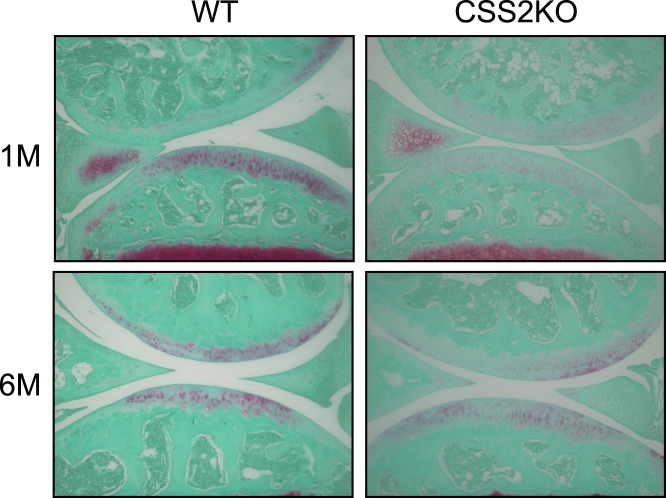
Histology of the articular cartilage of knee joints from CSS2^−/−^ (CSS2KO) mice and their wild-type (WT) littermates. Sections were stained with Safranin-O, which stains sulfated glycosaminoglycans, and fast green for counter staining. Each image shown is one representative section selected from 50 sections from an experimental group (10 sections from each knee joint and 5 knee joint in each experimental group). There were no obvious degradations of articular surface in knee joints shown and no morphologic differences among the groups at ages 1 and 6 months (1 M and 6 M).

CS is one of the major components of articular cartilage, and Csgalnact1-null mice exhibit some osteoarthritis-like changes in cartilage, such as a decreased level of aggrecan and link protein 1, a rapid catabolism of aggrecan, and abnormally aggregated and disarranged type-II collagen fibers [27], [28]. Contrasting to these mice, histological analysis of mouse knee joints at the age of 1 and 6 months using fast green and safranin O staining showed no articular degradation in any knee joints nor obvious morphologic changes in CSS2^−/−^ mice (Fig. 3).

### The Absence of CSS2 Affects the Chain Length, but not the Number and Sulfation Profiles of CS

**Figure 4:**
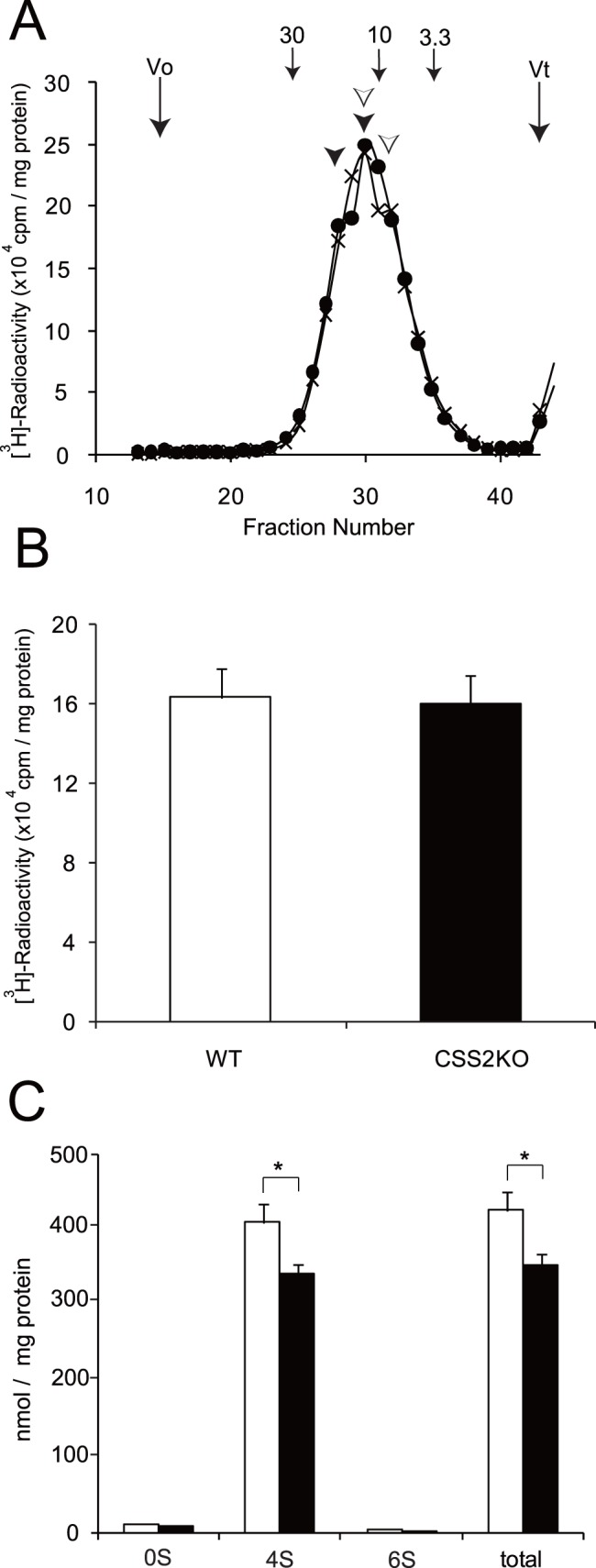
Structure of CS Chains in cartilage of newborn mice. *A*, CS extracted from proximal humeral cartilage of newborn mice was labeled with [^3^H]-sodium borohydride, applied to a Superose 6 column with effluent fractions of 0.5 ml each, and analyzed for radioactivity. The elution profiles of the samples obtained from CSS2^−/−^ (CSS2KO) (×) mice and wild-type (WT) (•) littermates are shown. Three independent experiments (n = 3) showed the same elution profile. *Numbered arrow 3.3*, *10*, and *30* indicate the eluted positions of chondroitin polysaccharides of known sizes (molecular size, 3,300, 10,000, and 30,000, respectively). *B*, Each total radioactivity of peaks of CSS2KO mice and WT littermates in Fig. 4A is shown. *C*, CS isolated from proximal humeral cartilage of newborn mice were digested with chondroitinase ABC and subjected to reverse-phase ion pair chromatography with postcolumn fluorescence labeling as described in “Experimental Procedures”. The histogram shows the amount and compositions of unsaturated disaccharide in the CS isolated from cartilage of CSS2KO (▪) mice and WT (□) littermates. All experiments were performed three times independently, and bars in the graphs were shown as a mean ± S.D. *, *p*<0.01. *0S*, *4S*, and *6S*, represent ΔDi-0S, ΔDi-4S, and ΔDi-6S, respectively.

Next, we performed biochemical analysis of CS. The chain length and disaccharide composition of CS were determined using proximal humerus cartilage of CSS2^−/−^ and WT newborn mice as described under “Experimental Procedures”. Gel filtration chromatography of CS samples obtained from cartilage revealed elution profiles with two peaks at fractions 28 and 30 for WT, representing molecular weight of 19,000 and 12,000, respectively, and two peaks at fractions 30 and 32 for CSS2^−/−^, representing that of 12,000 and 10,000, respectively (Fig. 4A). The total radioactivity quantified based on the elution profiles was similar between CSS2^−/−^ and WT (Fig. 4B). As the GAGs were labeled at the reducing end of individual chains, this result indicates that the number of CS chains was unaltered by the absence of CSS2. Analysis of disaccharide composition revealed a similar sulfation ratio of C0S (hexuronic acid-*N*-acetylgalactosamine, HexA-GalNAc), C4S (hexuronic acid-*N*-acetylgalactosamine-4-*O*-sulfate, HexA-GalNAc(4S)), C6S (hexuronic acid-*N*-acetylgalactosamine-6-*O*-sulfate, HexA-GalNAc(6S)), CSD (hexuronic acid-2-*O*-sulfate-*N*-acetylgalactosamine-6-*O*-sulfate, HexA(2S)-GalNAc(6S)) and CSE (hexuronic acid-*N*-acetylgalactosamine-4, 6-*O*-sulfate, HexA-GalNAc(4,6S)), representing 2.8%, 96.1%, 1.1%, 0% and 0% for WT cartilage, and 2.8%, 96.3%, 0.9%, 0% and 0% for CSS2^−/−^ cartilage (Fig. 3C). When the CS amounts were calculated based on the disaccharide composition data, the mass of CS in CSS2^−/−^ cartilage was 80% that in WT (Fig. 4C). When the same analysis was performed on brain, which also contains a large amount of CS, the CS amount in CSS2^−/−^ was ∼70% that of WT and the disaccharide composition was unaltered (data not shown). These results suggest that CSS2 is necessary for elongation of CS chains from approximately 10,000 in molecular weight, but it does not affect the number of CS chains.

### Expression of Other CSSs in the Absence of CSS2

**Figure 5:**
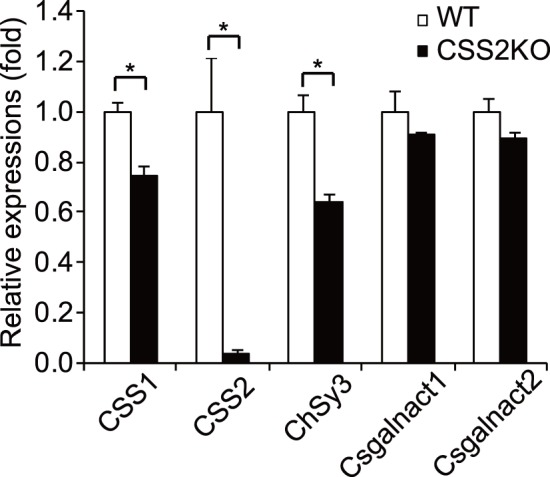
Expression level of CSSs in cartilage of CSS2^−/−^ (CSS2KO) mice and their wild-type (WT) littermates. Realtime RT-PCR using cDNA synthesized from humerus of newborn mice were performed to investigate relative expression levels of CSS1, CSS2, ChSy3, Csgalnact1, and Csgalnact2. The amount of expression was normalized by GAPDH. All experiments were performed three times independently, and bars in the graphs were shown as a mean ± S.D. *; p<0.05.

Although previous *in vitro* studies revealed that CSS2 was essential for CS synthesis [12], [13], [17], CSS2^−/−^ mice showed a certain level of CS polymerization and no effect on the number of CS chains. Therefore, we investigated whether other CSSs compensate for CSS2 function by up-regulation of their expression levels. When cDNA synthesized from proximal humerus of newborn mice was used as the template for quantitative realtime RT-PCR, the expression levels of CSS1 and ChSy3 in CSS2^−/−^ mice were significantly reduced to 75% and 65% that of WT mice, respectively, whereas those of Csgalnact1 and Csgalnact2 were not affected (Fig. 5). Though CSSs present in the Golgi apparatus may compensate for CSS2 function, these results confirm no compensation mechanisms at the transcriptional levels.

### CSS1 and CSS2 Differently Contribute to CS Biosynthesis

**Figure 6:**
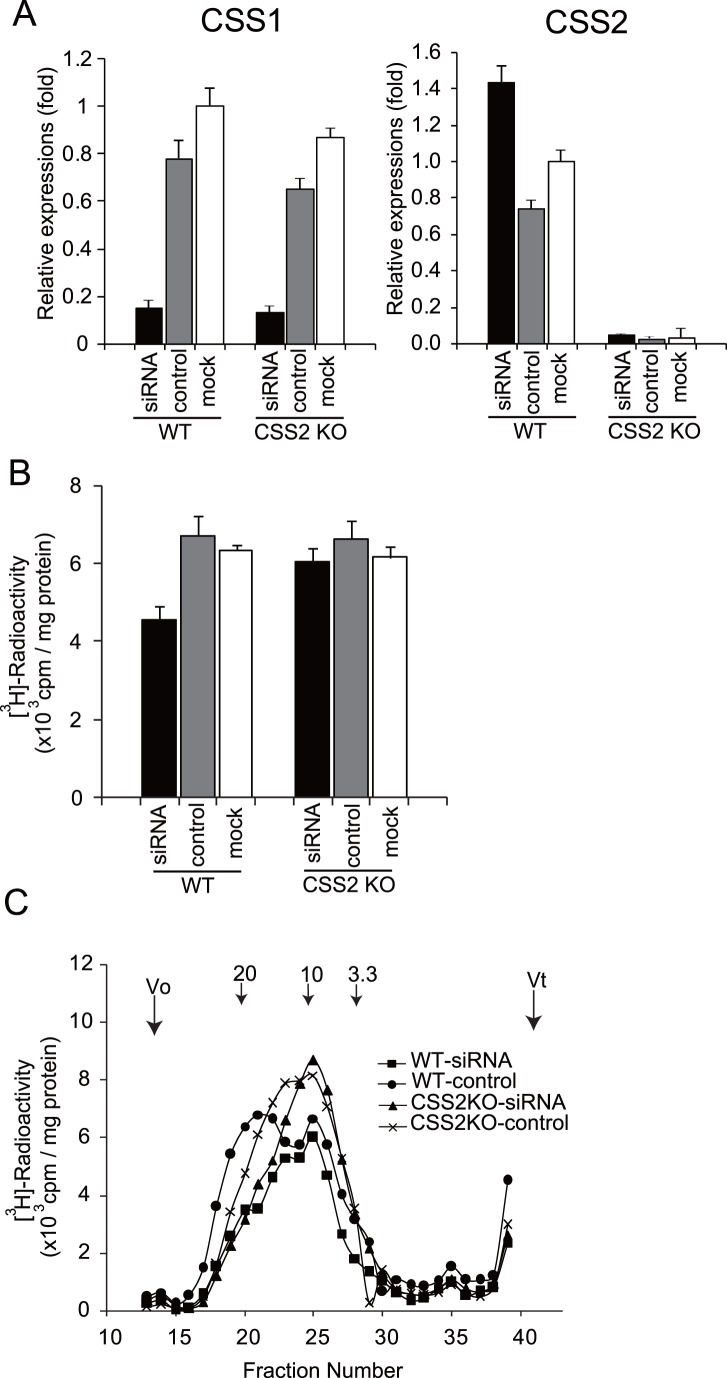
Effects of CSS1 siRNA in CSS2^−/−^ (CSS2KO) and wild-type (WT) chondrocytes on CS synthesis. Primary chondrocytes obtained from CSS2KO and WT mice were downregulated of CSS1 using siRNA, as described in “Experimental Procedures”. All experiments were performed three times independently, and bars in the graphs were shown as a mean ± S.D. *A*, Relative expression level of CSS1 and CSS2 in WT and CSS2^−/−^ chondrocytes were shown. siRNA, cells transfected with CSS1 siRNA; control, cells transfected with control siRNA; mock, untransfected cells. *B*, The total radioactivity of the elution profiles in each sample. *C*, Chain length of CS in chondrocytes. Three independent experiments (n = 3) showed the same elution profile. *Numbered arrow 3.3*, *10*, and *20* indicate the eluted positions of chondroitin polysaccharides of known sizes (molecular size, 3,300, 10,000, and 20,000, respectively).

Previous *in vitro* studies have revealed that the enzyme sample co-expressed with CSS1 and CSS2 exhibits the highest CS polymerizing activity, although that co-expressed with any two of four CSSs achieves polymerization [15], [17]. In the absence of CSS2, CSS1 may form a complex with another CSS and polymerize CS chains. Thus, we investigated the magnitude of contribution of CSS1 to CS biosynthesis in the presence and absence of CSS2. We cultured rib chondrocytes from WT and CSS2^−/−^ mice and transfected them with CSS1 siRNA. CSS1 siRNA and control siRNA downregulated CSS1 expression to ∼15% and 70∼80% in WT and CSS2^−/−^ chondrocytes, respectively, compared with mock transfectants when CSS2 expression was upregulated to ∼140% in WT chondrocytes by CSS1 siRNA (Fig. 6A). All of the siRNA controls and mocks of WT and CSS2^−/−^ chondrocytes showed the same level of total radioactivity of elution profiles, indicating the same number of CS chains in these cells. The total radioactivity of CSS1-downregurated WT chondrocytes showed ∼70% that of CSS1-downregulated CSS2^−/−^ cells (Fig. 6B), indicating that CSS1 affects the number of CS chains in the presence of CSS2. Next, we investigated the CS chain length in these chondrocytes. Whereas the elution profile of control siRNA in WT cells showed two peaks of 19,000 and 10,000 in molecular weight, CSS1 siRNA abrogated the peak at 19,000. Whereas the elution profile of control siRNA in CSS2^−/−^ cells showed two peaks of 12,000 and 10,000 in molecular weight, CSS1 siRNA abrogated the peak at 12,000 (Fig. 6C). Interestingly, the smaller molecular weight peaks of WT and CSS2^−/−^ (at 10,000) cells were unaffected by the downregulation of CSS1 or/and the absence of CSS2. These results suggest that CSS1 is involved in both CS chain initiation and polymerization, whereas CSS2 is involved only in the polymerization.

## Discussion

To date, six glycosyltransferases have been known to be involved in CS synthesis onto the linkage tetrasaccharide region. Biochemical studies have strongly suggested that a combination of CSS1 and CSS2 exhibits the most potent activity of CS synthesis. In this study, we generated CSS2^−/−^ mice and analyzed their phenotype. Although CSS2 was reported to be essential for CS biosynthesis [12],[13],[17], the mutant mice were viable and fertile, showing no overt abnormalities. Biological analysis demonstrated only a small reduction in the amount of CS in various tissues, which supports the absence of overt abnormalities in CSS2^−/−^ mice. Using chondrocyte culture systems, we investigated the function of CSS1 and CSS2, and have found that CSS1 participates in both the CS chain initiation and polymerization, and CSS2 participates in the polymerization, especially in further extension. Our results clearly demonstrate the distinct roles of CSS1 and CSS2 in the process of CS biosynthesis.

CSS2^−/−^ mice showed no overt abnormalities. Biochemically, cartilage and brain, which normally contain a large amount of CS, exhibited a decrease in it to 80% and 70%, respectively, in CSS2^−/−^ mice. These results indicate that CSS2 is not essential for development and growth, and that cartilage and brain are capable of CS synthesis up to at least 70∼80% without CSS2. Expression profiles of CS glycosyltransferases, though all are widely expressed in various tissues, have revealed that their expression levels are different among tissues. For example, CSS1 is expressed at high levels in lung, spleen, kidney, and liver, whereas CSS2 is expressed at high levels in heart, skeletal muscle, and brain [12], [13]. These observations suggest that there is a certain level of functional redundancy of CS glycosyltransferases *in vivo*, regardless of their expression patterns.

Various skeletal abnormalities with decreased CS have been reported. Gene trapped C4ST1-null mice, with ∼20% CS levels, die shortly after birth [26]. CSGalNAcT1-null mice [27], [28], with ∼50% CS levels in cartilage, exhibit slight dwarfism. They show some osteoarthritis-like changes in cartilage such as a decreased level of aggrecan and link protein 1, a rapid catabolism of aggrecan, and abnormally aggregated and disarranged type-II collagen fibers. Loss of function mutations of CHSY1 have been identified in human brachydactyly [29], . Recently, ChSy-1-null mice have been reported to exhibit profound limb patterning defect, similar to human mutations, with orthogonally shifted ectopic joints in distal digits [31], whereas CSS2−/− mice show no overt abnormalities as shown here. These observations support the notion suggested by biochemical analysis that ChSy-1/CSS1 has the most profound impact on CS chain biosynthesis. The absence of no overt skeletal phenotype in CSS2^−/−^ mice, including no osteoarthritic changes (Fig. 3), indicates that the threshold of CS levels for appearance of phenotype is 50∼70%.

A series of biochemical analysis have signified the role of CSSs in CS biosynthesis. Co-expression of any two glycosyltransferases out of CSS1, CSS2, CSS3, and CSGlcAT has been shown to exhibit CS polymerizing activity [15], [17]. Furthermore, the chain length polymerized by the co-expressed enzyme sample was different among the pairs. The enzyme sample co-expressed with CSS1 and CSS2 polymerizes the longest CS chains, followed by that of CSS2 and CSS3, and then that of CSS1 and CSS3, suggesting the selection of the pair determines the CS chain length [15]. We have noticed that there are at least two peaks of CS chain length in the elution profiles of gel chromatography. Indeed, the elution profiles of gel chromatography in this study (Fig. 3A and Fig. 6C) exhibit two peaks. This suggests that CS chain polymerization consists of two steps; i.e., a step from the initiation to elongation up to Mr ∼10,000, and a following step of further extension. Whereas the elution profile of CS obtained from WT chondrocytes contained two peaks at Mr ∼19,000 and 10,000, that from CSS2^−/−^ chondrocytes lacked the peak at Mr ∼19,000 and had peaks at Mr ∼12,000 and 10,000. As the chain number was unaltered, CSS2 may participate in the chain extension step. When CSS1 expression was inhibited by siRNA in CSS2^−/−^ chondrocytes, the peak at 10,000 became sharp and the peak at Mr ∼12,000 disappeared, indicating that CSS1 is mainly involved in the extension process in the absence of CSS2. When CSS1 expression was inhibited in WT chondrocytes, a similar elution profile to that of CSS2^−/−^ chondrocytes was obtained which lacked a peak at Mr ∼19,000. Interestingly, the CS chain number decreased to 70% (Fig. 6B), indicating that CSS1 is involved in both the chain initiation, as well as the extension. It is not clear why CSS1 exerts different functions in the presence and the absence of CSS2. CSS1 may normally form a complex with CSS2 for the chain extension and with another molecule for the chain initiation, and inhibition of CSS1 expression may affect both processes. In the absence of CSS2, CSS1 may totally participate in the chain initiation, and even if its expression is inhibited, residual levels of CSS1 may maintain the initiation process.

Several studies have attempted to inhibit CS synthesis by downregulating one of these CS glycosyltransferases. Decreased CSS3 expression to 60% with siRNA in HeLa cells has been shown a decrease in CS by 18% [15], and decreased CSS2 expression to 30% in astrocytes has achieved CS reduction to ∼40% [32]. However, our study has clearly demonstrated that the absence and/or down-regulation of two enzymes are required for adequate CS reduction. In this context, our CSS2^−/−^ mice are useful as the source of various cells for inhibition of CS synthesis, and for the generation of double and more knockout mice strains of CS glycosyltransferases.

CS contributes to water retention and therefore critical for the function of joint cartilage [33]. It is localized in the perineuronal net, which controls neuronal plasticity [34]. Accumulation of CS in the region of axonal injury inhibits neuronal regeneration, which can be restored by CS digestion with chondroitinase ABC [35]. Chondroitin is necessary for cell division of oocyte of C. elegans [36], [37]. Highly sulfated CS species have been shown to bind midkine and pleiotrophin, regulating their signaling [38]. Furthermore, recently, receptors of CS chains have been determined to be PTPsigma [39], NgR1 and 3 [40]. These accumulating results implicate the importance of CS chains in several biological and pathological processes. Our study may provide a clue to elucidation of the mechanisms of CS biosynthesis, and lead to manipulation of CS levels *in vivo*.
